# Efficiency of immediate postoperative inpatient physical therapy following total knee arthroplasty: an RCT

**DOI:** 10.1186/1471-2474-7-71

**Published:** 2006-08-31

**Authors:** Anton F Lenssen, Yvonne HF Crijns, Eddie MH Waltjé, Mike JA van Steyn, Ruud JT Geesink, Piet A van den Brandt, Rob A de Bie

**Affiliations:** 1 University Hospital Maastricht, Department of Physical Therapy, Maastricht, The Netherlands; 2 University Hospital Maastricht, Department of Physical therapy, Maastricht, The Netherlands; 3 University Hospital Maastricht, Department of Orthopaedics, Maastricht, The Netherlands; 4 Maastricht University, Department of Epidemiology, Maastricht, The Netherlands

## Abstract

**Background:**

The main goal of physical therapy treatment (PT) in the clinical stage following total knee arthroplasty (TKA) is to prepare patients for discharge from the hospital as soon as possible after their operation. Although aggressive rehabilitation is believed to be important, evidence of effects of different exercise programmes following TKA is limited. This led to the question whether the intensity of PT (once versus twice daily) following TKA affects short-term recovery, measured as range of motion.

**Methods:**

A randomised controlled trial compared an exercise regimen of two sessions per day with a similar programme administered once daily. Primary outcome measure was ROM.

**Results:**

At the time of hospital discharge, there was no difference between the experimental and control groups in range of motion.

**Conclusion:**

This study shows that in our setting twice daily PT sessions do not produce different results as daily PT sessions. It may be questioned whether multiple daily therapy sessions are needed as an in-hospital PT regimen in OA total knee patients.

## Background

Recent trends in the treatment of patients following orthopaedic surgery in the Netherlands encourage early discharge from hospitals, which implies increasing the intensity of treatment during the in-hospital phase. The strategy of choice involves mobilisation of the joints, starting on the day of surgery as well as shortening the bed rest phase and early ambulation. Physical therapy treatment (PT) plays an important role in this strategy.

In 2000 a 'Joint Care' programme was implemented at the University Hospital Maastricht. This is a clinical pathway in which the activities of physicians, nurses, physical therapists and other staff inside as well as outside the hospital are coordinated to provide the best overall care for patients with a particular diagnosis or procedure[1].

Clinical pathways are used in many institutions for patients undergoing total knee arthroplasty (TKA) and total hip arthroplasty (THA). These procedures lend themselves very well to a pathway approach, because the process of care is relatively standardized. Clinical pathways have been successful in reducing costs and length of stay in acute care hospitals, while not compromising patient outcomes [1-7]. The contents of the individual treatments are hospital-specific to create an optimal regimen of care tailored to that specific institution. The main goal in the clinical stage is to prepare patients for discharge from the hospital as soon as possible after their operation. Discharge depends primarily on restoring sufficient functional range of motion (ROM)[8].

Various authors [9-12] have described aggressive rehabilitation schemes incorporating more than one PT treatment session per day in the clinical phase following total knee surgery. Aggressive rehabilitation is believed to be important in preventing postoperative contracture of the soft tissue and in gaining better flexion. Nevertheless evidence of effects of different exercise programs following TKA is limited[13]. Our current PT programme is based on these rehabilitation schemes, as well as on the outcome of a nonrandomised controlled trial[14] comparing usual care with a 'Joint Care' clinical pathway. This trial found the length of hospital stay to be significantly shorter in the 'Joint Care' group. The author concluded that this was mainly caused by the greater stimulation to exercise and the number of minutes of PT per day. Patients were not only stimulated by their physical therapists to exercise, but the nursing staff also stimulated the patients to exercise and perform daily life activities themselves. Based on this result we doubled the time spent on in-hospital PT from 20 minutes to 40 minutes per patient per day, thus increasing treatment intensity to fulfil one of the goals of the programme; shortening hospital stay to four days after surgery. Patients are scheduled for discharge the fourth day after surgery if their passive flexion ROM exceeds 65°, when they are able to transfer from sitting to standing, walk independently, and are able to climb stairs, if necessary.

After two years of experience with the high intensity approach, the mean hospital stay was reduced to approximately four days after the operation. In 2003, however budget considerations meant that continuation of the extra PT sessions was being reconsidered. This led to a debate within the PT department whether or not the extra time investment should be maintained, i.e. whether the second daily visit was essential in reaching the short-term PT goals and whether having only one session of PT a day would lead to poorer range of motion (ROM) and function.

This resulted in the following research question. Does the intensity of PT treatment (once versus twice daily) in patients following TKA affect their short-term recovery, measured as range of motion?

To answer this question, a randomised controlled in-hospital trial was conducted in which a regimen consisting of four days with 40 minutes of PT a day was compared with one involving 20 minutes of PT a day.

## Methods

The medical ethics committee of the Maastricht University Hospital and Maastricht University approved this prospective, randomised controlled trial.

**Figure 1:**
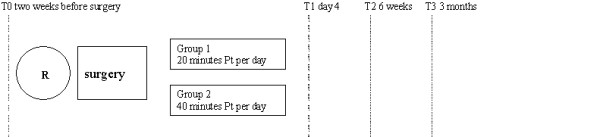
research design and outcome assessment. T0 baseline assessment, two weeks before surgery, T1 assessment 4 days after surgery, T2 assessment 6 weeks after surgery, T3 assessment 3 months after surgery. R = Randomisation.

The design of the trial is depicted in figure 1.

### Subjects

A consecutive series of patients who received a primary TKA at the University Hospital Maastricht, the Netherlands, was invited to participate between 1 January and 1 June, 2004. Subjects were considered eligible for the study if they were scheduled in the 'Joint Care' programme and signed an informed consent form.

Patients undergoing knee revision surgery were excluded as were patients over 85 years of age, patients with co-morbidity influencing gait and patients who did not understand or speak Dutch.

### Randomisation

An independent research assistant performed concealed randomization using a computer generated randomisation schedule in which patients were allocated according to a weekly treatment regimen. All patients are admitted into hospital on Monday evening, surgery is on Tuesdays and hospital leave is planned the next Saturday. By randomising clusters of treatment weeks instead of individual randomisation we made sure that patients within the same treatment week all received the same postoperative treatment regime, thus avoiding contamination, which might occur when patients receive different regimes in the same week of admission at the same ward.

Information on the particular treatment intensity was given on the day of surgery. In this way we prevented foreknowledge of treatment assignment and thus shielded those who enrolled participants from being influenced by this knowledge because enrolment into the study was conducted two weeks before surgery.

### Interventions

#### Clinical phase

During the in-hospital phase daily PT was administered to all participating patients. Patients in the experimental group were treated twice daily (totalling 40 minutes per day), patients in the control group once daily (20 minutes per day). The treatment content was similar in both groups and consisted of active and passive mobilisation of the knee, strengthening of the quadriceps muscle and functional exercises including transfers from a supine position to sitting and from sitting to standing, walking and stair climbing. As a rule, patients left hospital after the morning PT sessions on the fourth day after surgery, leading to a total number of PT sessions in the experimental group of seven sessions compared to four sessions in the control group.

A detailed description of the treatment protocol following total knee surgery is available at the website of the Physical Therapy department of the Maastricht University Hospital[15] We divided the treating physical therapists into two groups. One group treated all patients in the morning, while the optional second treatment sessions were administered by other physical therapists, leaving the group of therapists who treated the patients in the morning sessions unaware whether or not patients were receiving a second PT session. Therapist received weekly work schedules. During the study period therapist shifted from morning to afternoon groups randomly over treatment weeks. These changes were introduced to balance the work load of the participating therapist rather than for study purposes.

Hospital discharge followed when patients showed wound healing, they were ambulant using a walking frame or crutches and passive flexion ROM of the knee was above 65 degrees.

### Outcome measures

Primary outcome measure in this study was passive flexion ROM.

Secondary outcome measures were active ROM and passive extension ROM, functional status, length of stay (LOS) after surgery, pain over the last 24 hours (using an 11 point scale), satisfaction with treatment (using an 11 point scale from totally dissatisfied to completely satisfied)[16], satisfaction with the intermediate treatment results and global perceived effect.

ROM was measured actively as well as passively using a large goniometer following the method described by Brosseau[17]. Intratester reliability for knee flexion is 0.99, for active extension 0.97. Criterion validity for knee flexion 0.98, for extension 0.42[17].

Functional status was measured using two scales, the disease specific Western Ontario and McMaster University Osteoarthritis index (WOMAC)[18] and the joint specific Knee Society Scale (KSS)[19].

The WOMAC is a disease specific questionnaire developed specifically for people with osteoarthritis of the hip and knee. Using visual analog scales, its 24 items probe 3 dimensions – pain (5 items), stiffness (2 items), and functional difficulty (17 items). Scale sum scores were standardised (0–100), with high values indicating less pain or better physical functioning[20].

The WOMAC questionnaire is well recognized for its good validity, reliability and responsiveness. We used the Dutch version of WOMAC[20].

The KSS is concise and easy to use. It represents a clear attempt to separate knee function from overall patient functional status. Bach et al[21] reported that reproducibility of the knee score is poor whilst the function score shows good reproducibility. The construct validity of the KSS is good[22].

At follow-up, patients were asked to judge the effect of the surgery on a 7-point likert scale from 'worse than ever' status to 'completely recovered'[23].

The primary effect measurement was scheduled for the fourth day after surgery, while follow-up measurements were scheduled at 6 weeks and 3 months after surgery.

Table 1 shows the timing and measurement tools used. The outcome assessor was blinded with regard to the a clinical intervention regime administered.

We selected the KSS score as functional outcome on day four because several items in the WOMAC are not applicable during the in-hospital period. The WOMAC was assessed during the outpatient period because psychometric properties of the WOMAC are superior to the KSS.

### Sample size

A 10° of passive ROM difference between groups was considered clinically important enough to eventually raise treatment frequency. The sample size needed to detect a significant difference (2-sided, α <. 05) of at least 10° ROM between groups (primary outcome) with an assumed standard deviation (SD) of 10° and a power of 0.8, was 16 subjects per group. To compensate for a maximally acceptable 20% loss of subjects between baseline and follow-up, 20 subjects per group (40 in total) were required.

### Analyses

Data were checked for completeness and normality. Statistical analyses were blinded and performed according to the 'intention-to-treat' principle. Descriptive statistics were calculated of both group statuses at baseline. Post treatment scores as well as differences between pre- and post treatment scores were compared with regard to all outcome measures. Group differences and 95% confidence intervals (CI) were computed for all outcome measures. The scores on global perceived effect were dichotomized to examine the ratio of improved versus unimproved patients. Patients were scored as improved if they felt much better or had recovered completely. Student's t-test was used for continuous data to determine differences between the two treatment groups. Chi-square tests were used for analyses of categorical data and when continuous data were not normally distributed. All data were analysed using SPSS version 11.0[24].

## Results

Of the 55 subjects who were scheduled for surgery between January and June 2004, 43 were included in the study. 9 patients refused to participate and 3 were excluded because they had relevant co-morbidities. Twenty-one patients were randomly assigned to the experimental group, 22 to the control group.

The baseline characteristics of the patients in the two groups were similar in terms of clinical and demographic characteristics. They also showed comparable levels of functional ability and Quality of Life (table 2).

All 43 patients were assessed two weeks before surgery (T0) and four days after surgery (T1).

Six weeks after the operation (T2) 2 patients of the control group failed to attend the follow-up measurement, thus data from 41 patients were available for intention to treat analyses. All participants attended the follow-up measurements at 3 months.

### Outcome 4 days after surgery

As can be seen from table 3, there was no detectable difference in the primary outcome measure ROM between the experimental and control groups four days after surgery. Patients in the control group all received 4 treatment sessions. Two patients in the experimental group received six of the seven possible treatment sessions. One of these patients did not feel well enough to participate in a second PT session on the second day after surgery. The other patient left the hospital on the third day after surgery. The surgeon approved his discharge since he had reached the short-term treatment goals and asked to return home for personal reasons. Length of stay was similar in both groups. One patient in the control group was transferred to the cardiology department because of cardiac complaints. He left the hospital 15 days after surgery.

Flexion contracture was about 8° in both groups and active flexion ROM was about 70° Functional status results on day four were also similar between groups.

### Six-weeks and three-months follow-up

There were no significant or clinically relevant differences in the secondary outcome measures at any of the other follow-up moments (table 4). Follow-up data were comparable to those at day four after surgery. ROM in extension seemed to be a little better in the control group, as was satisfaction with the overall treatment. Recovery of flexion range of motion as well as functional status is similar in both groups.

## Discussion

This trial did not detect any differences in the effects on any of the outcome parameters between regimens involving one or two physical therapy sessions a day in the clinical phase following TKA.

Remarkably, patients seemed to be very satisfied with PT treatment, irrespective of the number of sessions. Three months after surgery, the perceived effect of the total treatment was high, with 39 out of 43 patients stating that they had vastly improved or were completely cured.

De Jong[14] reported similar numbers of PT sessions and comparable hospital stay (4.97 days) after surgery compared to our experimental group (4.1 days). De Jong did not report on ROM and functional capacity was measured using a scale of unknown reliability and validity.

Since we did not find any other studies on the effects of different PT treatment intensities following TKA, it is only possible to compare the results of the total group with those of other studies with known treatment intensity.

Most studies on rehabilitation after TKA have focussed on the use of continuous passive motion treatment.

Several recent studies [9,25-27] incorporated high intensity basic PT rehabilitation programmes and describe outcomes comparable to our data. Two studies employed standardized PT treatments twice daily, whilst Kumar et al. reported 90 minutes to two hours of PT daily and Beaupre et al. did not standardize the treatment duration but reported on six treatment sessions during the first four days after surgery.

Three [9,25,26] studies described short-term results on range of motion.

The ROM we found four days after surgery is comparable to the values reported in these studies. The level of pain found in our study is in agreement with those reported by Bennett whereas the length of stay in Bennett's sample was longer (8.8 compared to 5 days), and KSS scores at 3-months follow-up were slightly better in our sample (mean KSS function score of 52 versus 69).

Our study only collected Womac data preoperative and at six-weeks and three-months follow- up. No WOMAC data were collected at hospital discharge because several items of the WOMAC score are not valid during hospital stay, items like getting in and out of a car, shopping, getting in and out of a bath and doing domestic chores are not applicable in this phase.

### Study limitations

We chose cluster randomization of treatment weeks over individual randomization. By clustering in treatment weeks we made sure that all patients operated in a week were receiving the same amount of daily treatment sessions. We believe that this led to less bias than introduced by contamination in individual randomization. Patients receive PT treatment in the 'living room' of the orthopaedic ward in which they stay during daytime. We believe contamination is likely to occur when one patient would receive a second treatment in the afternoon whilst the patient sitting next to him/her is not. Conversation is overheard and just seeing treatment might encourage the patient to exercise them self, therewith diminishing contrast between both groups.

Though randomizing in clusters theoretically might inflict bias, we believed in this case it does not have a lot of influence. It is accepted that clustering may result in P values and confidence intervals which are sufficiently biased to have a major effect if any of the following are true: the cluster size is large, the number of clusters is small, or the intra-cluster correlation coefficient is large[28]. Our study contained 22 clusters, cluster sizes were small with a mean of two patients per cluster and intracluster correlation was very small, (icc = 0,171), leading up to a design effect of 1.171[29].

In combination with the fact that clusters were merely different weeks in a four months period in the same hospital with the same staff etc. makes us believe that clustering did not introduce a major bias in our study.

We chose passive flexion ROM as primary outcome measure because passive ROM is the only measure of function involved in hospital discharge in our situation. Therefore we based our sample size calculation on between group differences in passive flexion ROM.

When designing the study the therapists and orthopaedic surgeons involved discussed what effect would justify the effort of extra PT sessions daily. We had no information on possible effect sizes or minimally important clinical differences out of the literature, since we are unaware of a single similar study in this field. However we had some information on the effects of CPM in this same setting[30]. In this study we found an 8 degrees difference in flexion ROM when using CPM in the in hospital phase after total knee arthroplasty.

We agreed on a 10 degrees between group difference to be a result worth the extra PT effort. Although we did not detect a between group difference at all, one may state that our study was designed to find a large difference. Sample size was calculated to find this effect and therefore may be to small to detect smaller differences. Our study is underpowered to detect significantly smaller between group differences. For example, to detect a 5 degree difference sample size would a sample size of over 120 participants.

Although our study could not detect differences between groups receiving PT once or twice daily, this does not mean that individual patients might not benefit from more intensive PT. One of the primary issues in treating patients with TKA is to identify those patients that may require more intensive rehabilitation. Unfortunately, secondary analyses yielded no variables at baseline that predicted short-term postoperative recovery.

Different therapists treated patients in the morning and afternoon sessions as well as over different treatment days. This is a common procedure in our hospital which in itself might increase treatment variation. However, treatments were standardized and applied following a protocol.

In this study, PT treatment was standardized in terms of content as well as duration of the treatment sessions. Although almost every study in this field has done the same, one might question whether this is the appropriate way to handle patient care. It might be wiser to set treatment goals per day. As soon as the target for that day has been achieved no further treatment might be necessary. This would mean that the number of treatments can be individualized according to the goals set for each day.

Although blinding therapists is very difficult, we attempted to blind at least the physical therapists attending the morning session. Blinding was not successful in every case, because some patients gave information about the second session during the morning sessions.

Participating physical therapists stated that not knowing whether a patient would receive a second treatment session probably raised their awareness about achieving clinical goals during the morning sessions. Although they were aware that this attitude might bias study results, their attitude towards giving optimal patient care overruled the urge to follow the treatment protocol. In this sense one might say that treating patients once daily is just as good as twice daily if the physical therapists are more focussed.

## Conclusion

This study shows that in our setting twice daily PT sessions do not produce different results as daily PT sessions. It may be questioned whether multiple daily therapy sessions are needed as an in-hospital PT regimen in OA total knee patients.

## Competing interests

The author(s) declare that they have no competing interests.

## Authors' contributions

AFL participated in the design of the study, exercise intervention, assessments and follow-ups, statistical analyses and writing. YHFC, EMHW, MJAvS and RAdB participated in the study design and the interpretation of the data, and critically revised the article. RGTG and PAvdB participated in the interpretation of the data and critically revised the article. All authors read and approved the final manuscript.

## Pre-publication history

The pre-publication history for this paper can be accessed here:



## Figures and Tables

**Figure 2:**
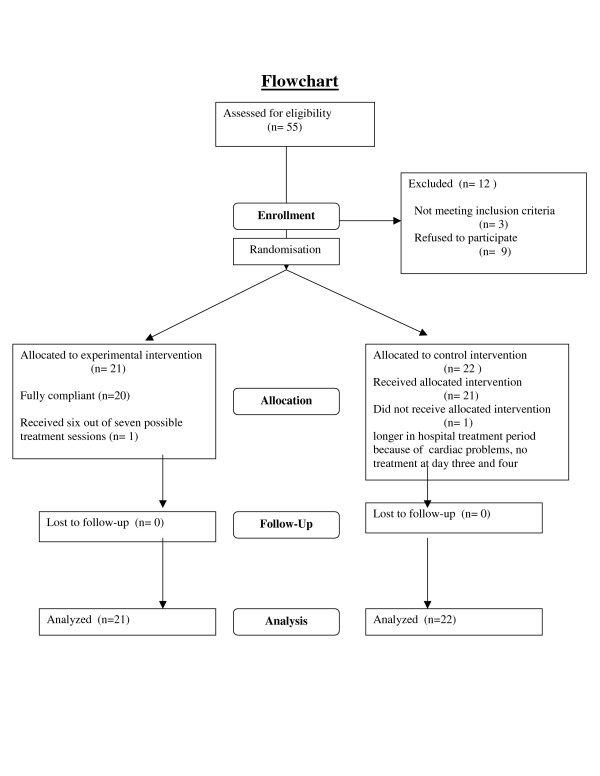
Flow chart of subjects through the trial.

**Table 1:** outcome measures and timing ROM = Range of Motion

	T0	T1	T2	T3
ROM	X	X	X	X
Functional status WOMAC	X		X	X
Functional status KSS	X	X	X	X
Length of stay		X		
Pain	X	X	X	X
Treatment satisfaction		X	X	X
Satisfaction with intermediate results		X	X	X
Number of PT sessions		X		
Global perceived effect of total treatment by the patient		X	X	X

T0, 14 days prior to surgery

T1, 4 days after surgery

T2, 6 weeks after surgery

T3, 3 months after surgery

**Table 2:** patient's baseline characteristics

Variable	Experimental group n = 21		Control group n = 22	
Female/Male	15/6		17/5	
	Mean	sd		
age	70	(8.5)	67	(7)
active ROM extension (degrees)	8°	(6°)	8°	(5°)
passive ROM extension (degrees)	5°	(5°)	5°	(5°)
active ROM flexion (degrees)	106°	(19°)	102°	(20°)
passive ROM flexion (degrees)	110°	(19°)	114°	(15°)
functional status WOMAC	44	(16.7)	40	(16.6)
WOMAC subscale pain	9.2	(2.7)	8.3	(3.5)
WOMAC subscale stiffness	3.7	(1.1)	3.3	(1.4)
WOMAC subscale difficulty	31.1	(9.3)	28.2	(11.8)
functional status KSS knee	49	(18)	43	(21)
KSS function	53	(18)	52	(21)
Pain (11 point scale)	3,7	(2,8)	3,5	(3,3)

**Table 3:** between group differences at day 4 after surgery (scheduled hospital discharge) T1 and differences between status 14 days prior to surgery and 4 days after surgery

Variable	Experimental group n = 21		Control group n = 22		Mean difference	95%CI	P-value	Differences between t0 and t1				Mean difference	95%CI	P-value
											
								Experimental group		Control group				
	Mean	sd	Mean	sd				Mean	sd	Mean	sd			
ROM active extension (degrees)	8.4	5.1	7.8	5.6	0.56	-2.7 – 3.8	0.73	0.71	8.6	0.14	5.2	0.57	-3.9 – 5.0	0.46
ROM passive extension (degrees)	5.5	4.5	5.8	4.9	-0.3	-3.2 – 2.6	0.81	0.1	7.2	0.4	4.7	-0.3	-4.2 – 3.2	0.78
Rom active flexion (degrees)	69.8	11.9	70.4	13.3	-0.5	-8.3 – 7.3	0.90	36.1	22.0	31.5	17.8	4.6	-7.8 -16.9	0.79
ROM passive flexion (degrees)	77.7	11.2	77.9	12.8	-0.2	-7.7 – 7.2	0.95	36.6	17.9	32.1	18.4	4.5	-6.8 – 15.6	0.43
Functional status KSS knee	54.3	15.2	52.2	17.8	1.1	-8.7 – 12.3	0.68	9.3	22.7	4.4	26.3	4.9	-20 – 10.2	0.52
Functional status KSS function	29.3	10.7	22.9	13.2	-6.4	-1.1 – 13.8	0.09	-24	23.4	-29	19.3	-5	-18 – 7.9	0.43
Length of stay	4.1	0.9	4.5	1.3	0.4	-0.3 – 1.0	0.34	-		-			-	-
Pain (11 point scale)	3.6	2.3	3.6	2.8	0	-1.6 – 1.6	0.97	-0.14	3.1	0.1	3.5	0.04	-1.8 – 2.3	0.82
Satisfaction with treatment (11-point scale)	9.1	0.8	9.1	0.7	0	-0.5 – 0.5	0.99	-		-			-	-

**Table 4:** Six weeks and three months follow-up

	6 weeks						3 months					
	Exp n = 21		Control n = 20		Mean difference	95%CI	Exp n = 21		Control n = 22		Mean difference	95% CI
	mean	sd	mean	sd			mean	sd	mean	sd		

ROM active extension (Degrees)	6.8	5.0	10.3	6.6	-3.5	-7.3 – 1.8	5.3	5.1	8.3	5.5	-3	-6.7 – 0.3
ROM passive extension (Degrees)	4.3	3.4	7.6	6.8	-3.3	-6.8 – 0.7	3.8	4.3	5.5	4.6	-1.7	-4.5 – 1.03
Rom active flexion (Degrees)	97.8	16.0	98	14.2	-0.2	-9.8 – 9.4	103.7	13	105.1	15	-1.4	-10.0 – 7.3
ROM passive flexion (Degrees)	102.6	16.4	102.5	14.2	0.1	-9.5 – 9.8	109	13	109.3	14.8	-0.3	-8.9 – 8.3
Functional status WOMAC	68.6	18.3	74.7	12.1	-5.9	-15.8 – 3.7	73.4	14.9	78.0	11.3	-4.6	-13 8 – 3.9
WOMAC subscale pain	14.2	3.9	15.6	2.5	-1.4	-3.4 – 0.69	15.2	3.0	16.2	2.4	-1	-2.7 – 0.7
WOMAC subscale stiffness	5.7	1.6	6.3	1.1	-0.6	-1.4 – 0.25	6.1	1.2	6.5	1.1	-0.4	-1.04 – 0.32
WOMAC subscale difficulty	48.6	12.9	52.9	8.4	-4.3	-11.2 – 2.5	51.9	10.6	55.3	8.3	-3.4	-9.2 – 2.5
Functional status KSS knee	71	18	77	13	-6	-16.1 – 4.1	80	17	80	18	0	-11.3 – 11.3
Functional status KSS function	60	14	58	18	2	-8.3 – 12.1	69	15	69	20	0	-11. -11.0
Pain (11-point scale)	1.8	2.5	2.4	2.5	-0.6	-2.2 – 0.9	1.3	1.9	0.8	1.5	0.5	-0.6 – 1.6
Satisfaction with treatment (11-point scale)	8.5	2.0	9.3	0.9	-0.8	-1.8 – 0.2	8.7	1.6	9.4	0.9	-0.7	-1.5 – .15
Perceived effect Improved/not	18/2		18/3				21/1		18/3			
